# A Review on Emerging and Reemerging of Infectious Diseases in Jordan: The Aftermath of the Syrian Crises

**DOI:** 10.1155/2018/8679174

**Published:** 2018-05-24

**Authors:** Nabil A. Nimer

**Affiliations:** Faculty of Pharmacy, Philadelphia University, Amman, Jordan

## Abstract

The review aims to examine the emergence and reemergence of infectious diseases in Jordan, in parallel with the Syrian refugee crisis. Qualitative approach has been adopted for systematically examining the outcomes of the Syrian crisis, which resulted in emerging and reemerging infectious diseases. It has adhered that infectious diseases, including measles, tuberculosis, and cutaneous leishmaniasis, have hazardous effects on Syrian refugees along with the local population in Jordan. The threat of major infectious diseases is higher and alarming in Jordan. National health policies should be implemented to adhere the influence of infectious diseases as well as to reduce the extent of infectious diseases in Jordan. In the 21st century, Syrian conflict can be deliberated as one of the biggest humanitarian disasters. In this multifaceted emergency with devastating requirements and limitations, it has been found essential for dominant medical healthcare providers to develop medical strategies that are based on comprehensive understanding of the concerned context and the main medical requirements and susceptible groups.

## 1. Introduction

In Syria, the healthcare system was comprised of a government-run public system prior to the conflict, which offered most of the primary care services. The private sector mostly focused on urban areas providing the majority of advanced care services. The past decades were categorized by the enhanced capacity of the healthcare system and swiftly developing national health indicators including the increased rate of child immunization and infant mortality rate. However, the commencement of civil war led to the worsening of the healthcare sector through the broad destruction of facilities, shortage of medicines, and healthcare personnel along with lack of secure transportation and secure routes [1].

Due to the instigation of the critical war situation in Syria, a massive upsurge of forced migration has been witnessed in March 2011. 4.8 million Syrian refugees migrated to the neighboring countries, which included Lebanon, Turkey, Egypt, Iran, and Jordan, for security and shelter [2]. The abrupt upsurge of refugee influx into these states has been a significant event that brought upon a severe negative impact on the world economy and ultimately generated strong economic and health pressures on Jordan [3].

The influence of infectious diseases among Syrians after the crisis is mainly attributed to the diverse factors that may include warfare and displacement of population, socioeconomic progression, poverty, human susceptibility, variation in accessibility, and quality of health care [1]. With the consideration of ongoing conflict in Syria as well as the nearby states, it is reflected that healthcare system in these zones has become one of the most defenseless and weak structures. It has been evaluated that healthcare facilities of Syria mostly involved the government public hospitals that focus and endow primary healthcare services, whereas private sectors are more inclined to provide advanced care services. The Syrian healthcare system has been incorporated throughout the civil warfare zone instead of endowing a secure region of health care and refuge [4]. Therefore, the study was aimed to examine the correlation between the Syrian crisis and the reemergence of infectious diseases in Jordan. The objectives primarily addressed the influence of Syrian conflict on infectious diseases, risks associated with the infectious diseases in Jordan, and status of infectious diseases in Jordan before the crisis, current situation, and future perspectives.

## 2. Methods

Concerning the objective, the study has entailed a qualitative research approach to examine the aftermath of the Syrian crisis on the population in the form of emerging and reemerging infectious diseases. Therefore, the review approach has been considered appropriate, concerning the nature of the study for in-depth analysis. 6.5 million Syrians have been displaced due to the ongoing three-year civil war [1], which left thousands of people killed or wounded by violence. It has developed a vacuum in the common infrastructures that may influence the regions throughout the years to come in future. Therefore, it is essential to review the war and its impact on the health of Syrian refugees after the conflict in Syria, causes of morbidities, and the emerging infectious diseases prevailing in Jordan and other countries.

The review included the information about viruses, bacteria, and parasites as provided in the reports from the WHO and Ministry of Health, Jordan. It mainly targeted the studies based on the outcomes of infectious disease in Jordan that also included the occurrence of infections in the state. The influx of Syrian refugees was also studied and correlated as it has presumed to be the main cause behind the prevailing rates of infectious diseases in Jordan. The study has explored peer-reviewed articles and reports generated by the World Health Organization in the context of infectious diseases, obtained from the web libraries of PubMed (January 2012 to November 2016), ScienceDirect (January 2014 to November 2016), and Elsevier (January 2013 to November 2016). Thirty-six peer-reviewed articles were searched on the basis of selected search terminologies. Different keywords have been utilized to search the most relevant articles. On the basis of abstract reading, some of the articles have been eliminated. Precisely, twenty-eight articles did not prove to be relevant to the specific systematic review study. Subsequently, eight of the total articles were chosen to be examined and evaluated.

The inclusion of the research content was entirely based on the quantitative and qualitative literature as well as the cross-sectional studies, related to the assigned keywords. The process of study selection included the processing of literature by initially reviewing the abstracts of the studies. Furthermore, the studies that presented with the significant information were selected for the analysis.

### 2.1. Review Based on Emerging and Reemerging of Infectious Diseases

In 2011, Syria descended into a civil war after the Arab Spring uprising. 13.5 million Syrians required humanitarian support as reported by the United Nations in March 2016. 13.5 million Syrian included 4.8 million people outside Syria and 6.6 million internally displaced individuals [5]. Presently, Turkey is hosting the greatest number of Syrian refugees that is around 2.7 million [5]. Very few refugees are living in camps around the border, and others may be spread in Turkey. The unpredictable and explosive increase in Syrian population living in Turkey had negative influences on the social and health determinants. The lack of healthcare opportunities has enabled shortages in childhood immunization plans and less availability of clean water and food provisions. Approximately 7.5 million Syrians were found at outpatient clinics according to Ministry of Health data and 299,240 were hospitalized [5]. The hospitalized Syrians were mostly wounded and injured victims, who have been occupying intensive care units. The refugees commonly live in unsanitary and uncrowded conditions, which may lead to the spread of skin, respiratory, genital, and gastrointestinal infections. There are still many problems pending to be resolved for better living and health standards.

Since 2014, an accelerated expansion in the number of global forced displacements has been observed. By now, the year has seen the highest record displacement in the history around the globe. It has been estimated that, by the end of 2014, about 59.5 million people were forced to migrate from their homes and displaced worldwide [6]. The movement primarily has been the result of conflicts, violence, and persecution in the region. Among the countries who provided shelters and security to the displaced population, Turkey is on the top of charts with accommodating around 1.59 million people, then Pakistan with 1.51 million people, Lebanon with 1.15 million [7], Iran with 982,000, Ethiopia with 659,500, and Jordan with 654,100 refugees [8].

The sudden upsurge of displacement to these states has brought upon major shifts in the economic and health paradigms. One of the primary concerns has been the declining health status in these regions. The rising numbers of infectious diseases in the parallel years of migration have been one of the main parts of the major demographic transitions in the areas. For achieving the objective, the literature has focused on the records of Jordan. The transition, as observed in the days of migration, displayed a high shift in the mortality rates and modifications in conventional lifestyles of Syrian refugees in Jordan [9]. Several studies revealed a pattern of diversity in the utilization of healthcare facilities as pursued by care-seeking individuals among the host Jordanians and refugees [10, 11]. The review further focuses upon the spread of infections and put an attempt to identify any existing correlations.

### 2.2. Disturbances Prevailed in Syria after Crisis

According to the World Health Organization (WHO), approximately 40% of ambulances and 57% of public hospitals were completely destroyed due to the persistent attacks in Syria [12]. Other studies have demonstrated that the healthcare services have been severely declining in an incessant manner [13]. Before the emergence of the Syrian crisis and influx of infectious diseases in the state of Jordan, preexisting infections within this region were considered to be the dominant factor contributing to mortality. The causes of death were made compulsory to be recorded by the Ministry of Jordan; however, information in regard to the causes of mortality was insufficient and erratic [14]. In recent decades, a significant contribution has been made to control the reasons behind prevailing mortality rate that included measles, influenza, and malaria. Subsequent to the proper preventive measures and management, these epidemics have disappeared as considerable variables, influencing the mortality status of Jordan, have been employed [15]. It has been estimated that 630,000 Syrian refugees crossed borders of Jordan in October 2015 [16]. It has been observed that government of Jordan is in its midphase to control the entrance of refugees and overcome its impacts. However, no possible solution has been lined up yet to resolve the major debt crisis [17]. The economic adversity in Jordan has been attributed to the decision taken in favor of Syrian refugees. The population influx generated diverse burden on the economy. Likewise, high burden of noncommunicable diseases also occurred with higher prevalence rates throughout the refugee camps [15].

### 2.3. Emerging Infectious Diseases among Syrian Refugees

The physical and mental health of the refugees is a challenging factor for the host countries. The displaced population indicated a high prevalence of chronic diseases as they tend to remain in a struggle for basic needs of life that primarily includes the healthcare facilities [18, 19]. The control of diseases among the refugees requires adequate attention to provide the population with needed treatment and management. Similarly, it has been perceived that the burden of infectious diseases can be eradicated from the secondary level facilities if they are endowed appropriately in the remote areas of Jordan [18].

Sharara and Kanj [1] examined the war and infectious diseases and studied the influence of circumstances of war on the prevalence of infectious diseases of global concern in a public health emergency. It has been notified that the poliomyelitis outbreaks have led the focus on the severity of the prevailing health issues internationally after the Syrian conflict. This attention also assisted to recognize the significance of reinvigorating elimination campaigns in the endemic nations including Nigeria, Pakistan, and Afghanistan for the prevention of such outbreaks. Polio has been declared as a public health emergency in Syria that needs international solidarity and efforts to avoid epidemic globally [1]. Similarly, Doganay and Demiraslan [5] studied the impact of reemerging infections, health services, and biosecurity in Turkey. In gunshot or surgical wounds, multidrug-resistant gram-negative bacterial infections appear to be a rising issue. Malaria, hepatitis A, and varicella have been seen with increasing incidence among the refugees. There are several issues, waiting to be resolved for living and health standards [5].

Several challenges have been reported in the field of infection control and management. To begin with, serious concerns have been observed in the clinical settings aligned with the causes of tuberculosis that focused on the control of the disease and reduction of multidrug-resistant tuberculosis (MDR-TB). Since 2013, an increase of 40% in the cases of tuberculosis has been adhered among Jordanian population, specifically among the Syrian refugees [20]. In the light of the recorded prevalence, it has been endowed to be essential for the children, as well as adults, to opt for proper screening and follow the probable detection measures at the initial stage of the disease [21]. El-Khatib et al. [22] has stated that the intensity of high care consumption of services among Syrian refugees, throughout Jordan, has notched a diverse illustration of infectious diseases along with the injuries. According to Doocy et al. [10], approximately 43.4% Syrian refugee individuals were detected with a type of chronic disease in 2015. Furthermore, the survey of UNHCR accounted that about 39.5% of individuals in Jordan were presented with chronic health status in the same year.

Cookson et al. [23] studied that the augmented extent of tuberculosis has adhered among internally displaced individuals throughout the Middle East and European regions. Cookson et al. [23] further demonstrated that the rates of tuberculosis were high among the displaced persons, but increased detection is predictable. Higher rates of tuberculosis were observed among the Syrian refugees and would predictably persist as the Syrian crisis endures. Active screening is probable to detect tuberculosis earlier for the reduction of risk for transmission. However, this strategy requires justifiable funding to continue, and there is a need to realize more activities. A consistent Jordanian tuberculosis strategy was developed for the Syrian refugees, which has potential to control efforts and inform treatment for other regional nations influenced by the Syrian crisis [23]. According to Wells, the higher incidence rate of tuberculosis has been observed to be constructed over time that turned out to adhere with the Jordanian populace [24]. Syrian refugees, living in the state of crisis, have been found to be suffering from restriction regarding the cure of tuberculosis. The main factor behind the limitation is the inadequate availability of medical facilities. The inadequacy consequently accounts for the decrease in the treatment and management of the disease [25]. Furthermore, the decrease in the detection of the disease and lesser notification from the population is another reason for the higher prevalence of the disease among the Syrian refugees in Jordan [24, 26].

Jaber et al. [27] stated that the prevalence of the infectious disease has been low in Jordan as compared to the northern parts of Syria. With the influx of Syrian refugees, the patterns of the disease incidence shifted and augmented the threat of emergence of the infection in Jordan. Similarly, the rate of the cases of tuberculosis presented an upsurge when the refugees from Syria were screened for the disease that ultimately generated a burden on the healthcare budget of Jordan [23] (Table 1).

### 2.4. The Impact of Armed Conflict on Health in Syria

A study by Hoetjes [28] examined the impact of armed conflict in Syria. The trust focused the direct and indirect impact of the war on the healthcare sector and results recognized the most susceptible groups and significant patterns in Syria. An adjusted framework was proposed based on conceptual model discussed the influence of armed conflict on health. Medical data demonstrated a rise in infectious diseases additionally to a significant existing burden of noncommunal diseases, nutritional problems, and mental health. These diseases prevailed among lactating and pregnant women, children under five years, and internally displaced population becoming more and more vulnerable in the existing context. The medical landscape has transformed and health-seeking options are becoming increasingly influenced by availability, security, and service affordability [28].

UNICEF [29] overviewed the overwhelming effects among children of three years of conflict in Syria. It has been reported that, prior to the conflict, the Syrian health profile comprised of a pressure of noncommunicable diseases. Since the conflict, it is suggested that the medical requirements are rising because of the additional influence of reemerging infectious diseases and high numbers of disabled and war-wounded individuals. The health system experiences a sequential breakdown and is consequently no longer able to state the health problems [29].

### 2.5. Causes of Morbidities in the Syrian Arab Republic

According to the data of Ministry of Health, the main causes of morbidities in 2006 are shown in Table 2. Digestive and respiratory diseases were the main causes for the people to search for health care.

### 2.6. Emerging Infectious Diseases in Jordan

Ismail et al. [30] reviewed the studies based on infectious diseases including polio, measles, and tuberculosis from 2005 to 2015. Communicable disease surveillance and control in the context of conflict and mass displacement in Syria have been focused. Infectious diseases have been reflected from manifold discrepancies among government and nongovernment regions. Development has been made to control disease surveillance, but immunization coverage endures to pose a threat to individuals of Jordan [30].

Doocy et al. [31] have highlighted the health service access and utilization among Syrian refugees in Jordan. A survey was conducted to assess the health-seeking attitudes and issues of Syrian refugees in Jordan. Cluster analysis along with the chi-square test and *t*-test methods was conducted. It was reported that the high incidence rate of cutaneous leishmaniasis, with the characteristics of spatial and temporal distribution, has emerged in the northern region, whereas 56.7% cutaneous leishmaniasis has been reported from Tartous along the northwestern parts of the country [23]. Doocy et al. revealed that almost 40% infants and children have been detected with rotavirus in 2016 [10]. According to Richardsen et al. [32], diarrheagenic *E. coli* strains were considered to be the second most usual cluster detected among enteropathogens. The major reason for the emergence of infectious diseases has been indicated by the conventional Jordanian practice of utilizing cooked foods and boiled milk in the diet [33]. The infectious diseases, correlated with clinical symptoms, are presented with the most prevalent enteropathogens observed in Jordan. The clinical characteristics and collective predictive capacity of temperature were investigated to determine the association among existence of pathogens, particularly the diarrheal pathogens [33]. It has been identified that due to the fact that the etiologic agents are not helpful in diagnosing a large number of diarrheal patients, the undiagnosed illness might get classified from the enteropathogens [32].

Almost 51.5% health-seeking services were sought from public sector facilities along with 9.7% in charity facilities. Care-seeking facilities were higher among Syrian refugees. Strategies are implemented to enhance the affordability and accessibility of health services for refugees and Jordanian individuals [31].

Assaf et al. emphasized that the devastated healthcare system has restricted immunization programs in Jordan, which consequently left thousands of individuals susceptible to the infectious diseases [34]. For instance, influenza vaccination is recognized as an effective way to prevent influenza and its problems [35]. However, there are many barriers identified between the modality of prevention and the general population. One of the main barriers among the Jordan population has been the fear of side effects, although the fear of the virus outbreak could have been the major reason to receive the vaccine. Nonetheless, as compared to other countries, the coverage rates have been lower in Jordan [34]. In Jordan, the majority of the households perceived the high cost of medical care as an obstacle to seek and receive standard care. Specialized care facilities and improved access to medications have been perceived to be among the primary requirements of the local population. It has been suggested that extra support to the poor households can prove to be beneficial in this regard [28].

It has been evaluated that approximately 60% of the Jordanian population live in urban areas. The health sector and Ministry of Jordan have been found influential in concentrating and reducing the roots of infectious diseases by considering its noteworthy impact on death status. In addition, a previous study has mentioned that prevalence and occurrence of harmful infectious diseases, including malaria and smallpox, are causing the negative influence on the individual's health [25]. It is observed that the prevalence of novel coronavirus has been described as a major cause of mortality in Jordan [3].

### 2.7. Emergence of Infectious Diseases in Other Countries

A great humanitarian emergency developed due to the conflict in Syria. Thousands of Syrians were killed, and more than half of the Syrian population migrated or were displaced after the Syrian conflict. Health care was vastly interrupted because of facilities' destruction and shortage of medical staff and medications. These circumstances created an appropriate condition that lead to the reemergence of polio, measles, cutaneous leishmaniasis, and tuberculosis. Jordan and Lebanon reported augmented tuberculosis rates among Syrian refugees. Incidents of cutaneous leishmaniasis were observed in Turkey, Lebanon, and Jordan other than Syria. After a long-term polio-free campaign in Syria, a polio outbreak was reported. Prevailing conditions of measles were accelerated due to the conflict. The healthcare facilities were overburdened in many countries that were hosting the immigrants of Syria including Lebanon, Turkey, Egypt, Iraq, and Jordan [36]. Unsanitary and crowded situations were also the reason of prevailing healthcare issues. It has been suggested that more supportive initiatives are required from international organizations for the diagnosis, treatment, and control of the infectious diseases that are prevailing after the conflict in Syria.

It has been concluded that, without security, there can be no health satisfaction. All efforts in Syria to rebuild the destroyed healthcare sector and quell humanitarian disasters will be greatly ineffective as long as the civil war endures to rage on. The end of war immediately is complex from efforts to control the global threat and spare the innocent lives from the infectious diseases. Healthcare circumstances are uncontrolled by the geopolitical borders, while the political borders of the conflict can be allocated. The spread of communicable diseases and refugees into Jordan, Iraq, and Lebanon defines the prevailing consequences of the extended Syrian conflict. Additionally, the infections and diseases continue to influence the increasingly vulnerable population negatively. The international community has been insufficiently observed in response to the consequences of infectious diseases after the Syrian conflict. This failure may continue to increase until there are exhaustive and coordinated global efforts.

The study has contributed to identify an important issue on the current global problem of refugees, especially due to the conflicts added by war crimes. It has been examined that advanced strategies have been developed to control the augmenting numbers of the infectious diseases. Major progress has been perceived in the healthcare services, while governance and coordination issues have been found to be on the verge of exposing the local populace in Jordan to the risk of contamination. The threat of major infectious diseases has been higher in extent as well as threatens an alarming medical situation in Jordan. Furthermore, the cost has been identified as a considerable constraint to the healthcare services in Jordan in spite of high levels of care-seeking. Development must also be made to control the disease surveillance along with the immunization coverage. Further strategies must be implemented to develop national policies and strong health promotion services to highlight prevention of infectious diseases among both the local population and sheltered refugees within the borders of the state.

## Figures and Tables

**Table 1:** Review analysis.

Author	Title	Methodology	Conclusion
Sharara and Kanj [1]	War and Infectious Diseases: Challenges of the Syrian Civil War	This study was conducted to evaluate the effects of Syrian crises on the spread of measles, poliomyelitis, and cutaneous leishmaniasis. Data of Syrian refugees and civilians from Syria and the region were examined.	It was revealed that although there was a slow and consistent spread of infectious diseases, relevant governments were taking measures to contain them. The Lebanese government's campaign included free medical treatment and spraying pesticides to kill the sand vector that caused cutaneous leishmaniasis.

Doganay and Demiraslan [5]	Refugees of the Syrian Civil War: Impact on Reemerging Infections, Health Services, and Biosecurity in Turkey	Out of a total 13.5 million Syrian refugees and internally displaced persons, 2.7 million were being hosted by Turkey. The study examined the prevalence and spread of infectious diseases such as measles, poliomyelitis, leishmaniasis, and multidrug-resistant tuberculosis along with other diseases among Syrian refugees in Turkey.	It was recorded that measles, poliomyelitis, leishmaniasis, and multidrug-resistant tuberculosis were the reemerging infections. Moreover, gunshot wounds showed multidrug-resistant gram-negative bacterial infections. Other diseases such as malaria, hepatitis A, and varicella were most commonly diagnosed in refugees too.

Skye El Sayegh and Hussein [13]	Overview of Some Emerging Infectious Diseases: A New Era for New Vaccines	This study investigated the prevalence of infectious diseases such as dengue, Ebola, and Middle East respiratory syndrome coronavirus among Syrian refugees in Middle Eastern region. It also included discussion on their origin, mode of transmission, and widespread effects on the general population. Furthermore, it examined the status of vaccines being developed to control the spread of these diseases.	It was concluded that these diseases bore a huge health and economic burden with annual costs of almost a billion dollars with 70,000 hospitalizations, 200,000 ED visits, and 20 to 60 deaths in children below the age of 5 years. These diseases pose a major threat to the economy as well as the public health systems around the world. The importance of vaccines cannot be overstated in such dire situations.

Petersen et al. [14]	Infectious Disease Risk from the Syrian Conflict	In the study, a health report by ProMED and Medecins Sans Frontieres had been evaluated and examined with regard to the prevalence and spread of diseases such as rabies, MERS-CoV, upper respiratory tract, gastrointestinal, and skin infections, measles, and other vaccine-preventable infections.	Syrian refugees were having tuberculosis, leishmaniasis, and brucellosis. Different epidemics such as gastrointestinal infections and bacterial meningitis were also found to be prevalent, and there was a major risk of these diseases spreading among the population.

Hoetjes [28]	The Impact of Armed Conflict on Health in Al-Raqqah Governate, Syria	The study examined medical data to determine the effects of war on health of vulnerable groups of people in Al-Raqqah governorate in Syria.	The study concluded that, along with the existing burden of increasing prevalence and spread of infectious diseases, other health problems were also prevalent due to the war. It was recommended that the primary health care (PHC) must be strengthened and expanded to safe areas around Al-Raqqah governorate. Additionally, referrals must be made to specialized healthcare hospitals that are located in stable areas. Medical interventions necessitate sustainable connections within a community.
Ismail et al. [30]	Communicable Disease Surveillance and Control in the Context of Conflict and Mass Displacement in Syria	A peer-reviewed and non-peer-reviewed literature was conducted. Apart from this, a secondary analysis of monitoring data from early warning systems was performed on three diseases measles, polio, and tuberculosis.	It was concluded that disruption to critical health infrastructures, lack of laboratory services, and poor vaccination coverage created a risk of controlling the spread of communicable and noncommunicable diseases. All these problems were also due to poor coordination, poor access, and other security problems.

Ozaras et al. [36]	The Syrian Conflict and Infectious Diseases	The study examined the reemergence of polio, measles, tuberculosis, and leishmaniasis in Lebanon, Jordan, Syria, and Turkey after the Syrian crisis.	It was reported that, after a 15-year gap, Syria faced a polio outbreak. Outgoing measles outbreak was also accelerated. Additionally, Jordan and Lebanon were reported to have increased rates of tuberculosis among Syrian refugees.

Jaber et al. [27]	An Exploratory Comparative Study of Recent Spatial and Temporal Characteristics of Cutaneous Leishmaniasis in the Hashemite Kingdom of Jordan and Syrian Arab Republic Pre-Arab Spring and Their Health Policy Implications	The retrospective study examined the spatial and temporal characteristics of cutaneous leishmaniasis in Syria and Jordan.	The results concluded that the patterns of cutaneous leishmaniasis disease were low and more heterogeneous in Jordan. Conversely, patterns of cutaneous leishmaniasis disease were relatively much higher and less heterogeneous.

**Table 2:** Causes of morbidity in the Syrian Arab Republic.

Causes of morbidity	
Diseases	Percentages
Digestive diseases	15.7
Respiratory diseases	13.2
Cardiovascular diseases	11.3
Infectious and parasitic diseases	6.9
Blood diseases	3.2
Accidents	2.9
